# Temporal Expression Patterns of Genes Related to Sex Steroid Action in Sexually Dimorphic Nuclei During Puberty

**DOI:** 10.3389/fendo.2018.00213

**Published:** 2018-05-02

**Authors:** Moeko Kanaya, Masahiro Morishita, Shinji Tsukahara

**Affiliations:** Division of Life Science, Graduate School of Science and Engineering, Saitama University, Saitama, Japan

**Keywords:** aromatase, puberty, sex steroids, sexual differentiation, sexually dimorphic nucleus, sex steroid receptor, anteroventral periventricular nucleus, principal nucleus of the bed nucleus of the stria terminalis

## Abstract

Sex steroids play a major role in sexually dimorphic brain development during not only the perinatal period but also the pubertal period. We previously showed that, in male mice, the estrogen receptor-α (*Esr1*) and aromatase (*Cyp19a1*) genes are essential to the sexually dimorphic formation of the anteroventral periventricular nucleus (AVPV) and the principal nucleus of the bed nucleus of the stria terminalis (BNSTp), but the estrogen receptor-β (*Esr2*) gene is not necessary. We also showed that the androgen receptor (*Ar*) gene is essential to the sexually dimorphic formation of the BNSTp. These genes are expressed in the AVPV and BNSTp of perinatal mice. However, it remains unknown whether these genes are expressed in the AVPV and BNSTp during puberty, and whether the expression, if any, differs by sex, age, and brain region. Here, we dissected the AVPV and BNSTp from Nissl-stained brain sections of male and female mice on postnatal day (PD) 20 (prepuberty), PD30 (puberty onset in females), PD40 (puberty onset in males), and PD60 (young adult) using a laser microdissection system. We then examined the mRNA levels of *Esr1, Esr2, Cyp19a1*, and *Ar* in these brain regions. In the AVPV, *Esr1* mRNA levels were greater in females than males during PD20–60. *Esr2* and *Ar* mRNA expressions did not differ between sexes. *Ar* mRNA levels were higher at PD30 than PD20. *Cyp19a1* mRNA was not detected in the AVPV at PD20–60. In the BNSTp, *Esr1* and *Esr2* mRNA levels were higher in females than in males during PD20–60, although the mRNA levels of *Cyp19a1* and *Ar* did not differ between sexes. Additionally, we revealed that orchiectomy at PD20 induced a failure of normal formation of the male BNSTp and testosterone replacement in the prepubertal period rescued the effect of orchiectomy at PD20. Taken together, it is suggested that pubertal testosterone transported to the AVPV is not converted to estradiol there and does not act *via* ESR1 and ESR2. By contrast, the formation of the male BNSTp may be affected by testicular testosterone during puberty *via* AR and/or *via* ESR1 after conversion to estradiol by CYP19A1.

## Introduction

Sexually dimorphic nuclei underlie sex- and gender-specific functions in the brain. The anteroventral periventricular nucleus (AVPV) of the hypothalamus in rodents is a sexually dimorphic nucleus that is larger in size and contains more neurons in females than in males (1–4). Specifically, the female AVPV contains a larger number of neurons expressing tyrosine hydroxylase (TH) (3) and kisspeptin (5, 6). TH neurons in the AVPV promote parental behavior in female mice (7). Although TH neurons in the AVPV do not promote parental behavior in male mice, they suppress inter-male aggression (7). Kisspeptin neurons in the female AVPV appear to participate in the estradiol-induced surge of luteinizing hormone (8, 9). The principal nucleus of the bed nucleus of the stria terminalis (BNSTp) is a sexually dimorphic nucleus in the forebrain that is larger and contains a greater number of neurons in male rodents (10–13). Males have a greater number of vasopressin neurons in the BNSTp, which are involved in anxiety, aggressive behavior, and stress responses (14), and which project to the lateral septum (15–17). These differences in vasopressin neurons may underlie behavioral sex differences, particularly as male rodents exhibit more anxiety-related behavior than females (18, 19).

In male rodents, sexually dimorphic nuclei are formed by the influence of testicular testosterone during the perinatal period (20, 21). Like the AVPV in male rats, the AVPV in female rats treated with testosterone postnatally is smaller and contains fewer TH neurons and kisspeptin neurons in adulthood (6, 22). We previously showed that the volume and number of neurons in the AVPV are increased in male mice with deletion of the estrogen receptor-α (*Esr1*) and aromatase (*Cyp19a1*) genes, but are not affected by deletion of the estrogen receptor-β (*Esr2*) or androgen receptor (*Ar*) gene (23). These previous findings suggest that *Esr1* and *Cyp19a1* are essential to the formation of the male AVPV, but *Esr2* and *Ar* are not necessary. In support of this notion, *Esr1* and *Cyp19a1* mRNAs are expressed in the AVPV in perinatal mice (23). Postnatal treatment with estradiol, as well as testosterone, reduces the number of neurons in the AVPV in adult female rats (24). Thus, testicular testosterone in the perinatal period might be converted by CYP19A1 in the AVPV to estradiol, which signals through ESR1 to defeminize the morphology of the AVPV.

Perinatal testicular testosterone also affects the BNSTp, although the effects are opposite to those on the AVPV. The volume and number of neurons in the BNSTp in adult male rats are decreased by neonatal orchiectomy (12). Postnatal treatment with testosterone or estradiol increases the number of neurons in the BNSTp of adult female mice (25). The volume and number of neurons in the BNSTp in male mice are reduced to levels similar to those in females by deletion of *Esr1* or *Cyp19a1*, but not *Esr2* (26). *Esr1* and *Cyp19a1* are expressed in the BNSTp in perinatal mice (23). These findings suggest that the formation of the male BNSTp involves the action of estradiol (produced by the aromatization of testosterone) *via* ESR1 during the perinatal period. Additionally, the AR plays an important role in the formation of the male BNSTp, because the volume and number of neurons in the BNSTp are smaller in male *Ar* knockout (KO) mice, compared with wild-type males, similar to females (23). *Ar* is not expressed in the murine BNSTp prenatally, but begins expression early in the postnatal period (23, 27). The formation of the male BNSTp might require not only estradiol, formed from the aromatization of testosterone, acting *via* ESR1 in the perinatal period, but also testosterone acting *via* AR postnatally.

Accumulating evidence shows that testicular testosterone during the perinatal period is essential for the sexually dimorphic formation of brain structures. However, the formation of sexually dimorphic nuclei is affected by pubertal gonadal hormones as well (28, 29). In male mice, the BNSTp contains a comparatively greater number of calbindin neurons (30). This sex difference in calbindin neurons emerges before puberty, and increases further as calbindin neurons continue to increase in males and decrease in females during the pubertal and adolescent periods (31, 32). Testicular hormones during puberty also contribute to the formation of the male BNSTp, because the increase in calbindin neurons in the male BNSTp is perturbed by prepubertal orchiectomy, although the decrease in the female BNSTp is not altered by prepubertal ovariectomy (32). The sexually dimorphic formation of the AVPV might be influenced by pubertal ovarian hormones. Prepubertal ovariectomy decreases the volume and number of neurons in the AVPV in female rats (33). Thus, gonadal hormones during puberty play an important role in the sexually dimorphic formation of the AVPV and BNSTp; however, it remains unknown whether genes related to gonadal hormone actions are expressed in the sexually dimorphic nuclei during puberty.

In this study, we aimed to determine whether the AVPV and BNSTp of pubertal mice express genes involved in gonadal hormone actions, and whether the expression, if any, varies by sex, age, and brain-region. First, using tissue samples isolated precisely and accurately from the AVPV and BNSTp of peripubertal mice, we evaluated the mRNA expression patterns of *Esr1, Cyp19a1*, and *Ar*, which play an essential role in the sexually dimorphic formation of the AVPV and/or BNSTp (23, 26), as well as that of *Esr2*. As a result, the mRNA of *Cyp19a1* was expressed in the BNSTp, but not in the AVPV of peripubertal mice, suggesting that the BNSTp of male mice is affected by estradiol, which is converted from testicular testosterone by CYP19A1 locally there during puberty, but the AVPV is not. Next, to determine whether testicular testosterone during puberty contributes to the formation of the male BNSTp, we investigated the effects of prepubertal orchiectomy and testosterone replacement on the morphology of the BNSTp in adulthood with reference to calbindin expression.

## Materials and Methods

### Animals

Adult male and female C57BL/6J mice for breeding were purchased from Sankyo Labo Service Corporation (Tokyo, Japan). Offspring derived from mating in our facility were housed with dams in the same cages until weaning on postnatal day (PD) 21 (PD0 = day of birth). All animals were bred and housed in a room with a controlled temperature (22°C) and a 12-h light/12-h dark cycle (lights on: 08:00–20:00). Standard diet and tap water were available *ad libitum*. All animal experimental procedures were approved by the Animal Care and Experimentation Committee of Saitama University and were conducted in accordance with the Guidelines for the Care and Use of Experimental Animals of Saitama University.

### Gene Expression Analysis of the AVPV and BNSTp in Peripubertal Mice

#### Brain Tissue Collection

Male and female mice of different ages {PD20, prepuberty; PD30, puberty onset in females; PD40, puberty onset in males; and PD60, young adult [see review by Piekarski et al. (34)]} were deeply anesthetized by intraperitoneal injection of sodium pentobarbital (64.8 mg/kg body weight) and decapitated. Fresh brains were quickly frozen in hexane chilled to −80°C and stored at −80°C until further processing. There are several studies showing that the levels of *Esr1* mRNA and protein in the preoptic area (35, 36) and the number of ESR1- and AR-immunoreactive cells in the BNSTp (37, 38) are affected by circulating estradiol. Therefore, we compared the mRNA levels of *Esr1, Esr2, Cyp19a1*, and *Ar* in the AVPV and BNSTp of diestrous and proestrous female mice. When collecting brains from females on PD40 or PD60, vaginal smears were performed starting on the day of vaginal opening, and animals in the diestrous or proestrous phase were sacrificed between 12:00 hours and 14:00 hours on PD40 or PD60. As a result, unexpectedly, diestrous and proestrous female mice did not differ with regard to the mRNA levels of *Esr1, Esr2*, and *Ar* in the AVPV and BNSTp (data not shown). *Cyp19a1* mRNA was not detected in the AVPV on PD40 and PD60. *Cyp19a1* mRNA in the BNSTp did not differ between diestrous and proestrous females on PD40 and PD60 (data not shown). In this study, diestrous and proestrous female mice of the same age were therefore combined into a single group. The numbers of animals used for this study were as follows: PD20 male, *n* = 6; PD20 female, *n* = 4; PD30 male, *n* = 4; PD30 female, *n* = 5; PD40 male, *n* = 4; PD40 female, *n* = 6; PD60 male, *n* = 4; PD60 female, *n* = 8.

#### Isolation of the AVPV and BNSTp

The AVPV and BNSTp were dissected using a laser microdissection (LMD) system (Leica LMD 7000; Leica Microsystems, Wetzlar, Germany) in accordance with the procedure reported previously, with slight modification (23). Briefly, frozen brains were coronally cut at a thickness of 30 µm on a cryostat. Brain sections containing the AVPV and BNSTp were obtained in the coronal plane 0.38–0.02 mm rostral to the bregma and 0.10–0.34 mm caudal to the bregma, respectively, according to the mouse brain atlas (39). Brain sections containing the AVPV or BNSTp were mounted on PEN membrane slides (Leica Microsystems), fixed with ice-cold 5% acetic acid in ethanol for 3 min, stained with ice-cold 0.2% cresyl fast violet for 1 min, rinsed in 100% ethanol for 1 min, and dried with cool air. The AVPV and BNSTp were then dissected out from cresyl fast violet-stained brain sections by the LMD system and collected in a tube containing 70 µL RNA extraction buffer and 5 µL carrier RNA working solution (4 ng/µL) from the RNeasy Micro Kit (Qiagen, Valencia, CA, USA). To confirm whether the AVPV and BNSTp were precisely collected, we measured the volume of the nuclei. If the AVPV and BNSTp were correctly isolated, a sex difference in the volume of the nuclei could be found. The areas of the dissected tissue fragments were recorded by the LMD system. We calculated the volume of the acquired tissues by multiplying total of the areas of the dissected tissue fragments by the thickness of brain sections (30 µm) for each sample.

#### Quantification of mRNA Levels

Total RNA was extracted and purified using the RNeasy Micro Kit (Qiagen) according to the manufacturer’s protocol. First-strand cDNA was synthesized using the TaKaRa Prime Script RT reagent kit (TaKaRa Bio, Otsu, Japan). For each sample, total RNA (approximately 160 ng) was reverse-transcribed to first-strand cDNA in a final volume of 15 µL Prime Script buffer containing Prime Script RT Enzyme Mix I (0.75 µL) and random hexamers (75 pmol). Standard samples for *Esr1, Cyp19a1, Ar*, and glyceraldehyde-3-phosphate dehydrogenase (*Gapdh*), a housekeeping gene, were prepared by mixing an equal amount of each cDNA sample and serially diluting in EASY Dilution solution (TaKaRa Bio). To prepare standard samples for *Esr2*, cDNA obtained from the hypothalamus of adult mice was serially diluted.

Quantitative polymerase chain reaction (qPCR) was performed using a Light Cycler ST300 (Roche Diagnostics, Mannheim, Germany). A 2-µL aliquot of standard or unknown sample was amplified in a 20-µL reaction mixture containing 200 nM of each gene-specific primer [*Gapdh* forward primer: CACTGCCACCCAGAAGA, *Gapdh* reverse primer: TCCACGACGGACACATT; other genes were designed according to our previous study (23)] and 10 µL of 2× SYBR Premix Ex Taq (TaKaRa Bio). The thermocycling parameters for qPCR were 30 s at 95°C, followed by 40 cycles of 95°C for 5 s and 60°C for 20 s. After qPCR, melting curve analysis was performed to assess the specificity of the PCR products. This analysis showed that the melting curves for the PCR products all had a single peak (data not shown).

qPCR analysis for *Gapdh* mRNA in the AVPV and BNSTp revealed that *Gapdh* mRNA levels in these nuclei did not differ significantly by sex or age (data not shown). Therefore, the amount of *Esr1, Esr2, Cyp19a1*, and *Ar* mRNAs was normalized by dividing by the amount of *Gapdh* mRNA for each sample.

### Morphological Analysis of the BNSTp in Male Mice

#### Prepubertal Orchiectomy and Testosterone Replacement

At PD20, male mice were orchiectomized and subcutaneously implanted with a Silastic tube [1.98 mm inner diameter, 3.18 mm outer diameter, 10.9 mm in total length (effective length: 6.9 mm); Dow Corning Corporation, Midland, MI, USA] containing testosterone (Sigma-Aldrich, St. Louis, MO, USA; *n* = 5) or cholesterol (Wako Pure Chemical Industries, Osaka, Japan; *n* = 5). The testosterone implantation was designed to produce the levels of serum testosterone in young male mice (Tsukahara, unpublished data). Surgery was performed under isoflurane inhalational anesthesia (concentration, 1.5% in air; flow rate, 1 L/min). Additionally, we prepared gonadally intact males (*n* = 5).

#### Tissue Preparation

At 10 weeks of ages, all animals were deeply anesthetized by intraperitoneal injection of sodium pentobarbital (64.8 mg/kg body weight) and transcardially perfused with 0.05 M ice-cold phosphate-buffered saline (PBS; pH, 7.4) followed by ice-cold 4% paraformaldehyde in 0.05 M phosphate buffer (pH, 7.4). Brains were postfixed with the same fixative at 4°C overnight and then immersed in 30% sucrose in 0.05 M phosphate buffer at 4°C for 2 days. Fixed brains were quickly frozen, coronally sectioned at a thickness of 30 µm using a cryostat, and collected at 60-µm intervals.

#### Immunohistochemistry for Calbindin

Brain sections were rinsed in 0.05 M PBS containing 1% Triton X-100 (PBST) and placed in 0.6% H_2_O_2_ in PBST for 30 min at room temperature. The sections were then placed in 5% normal goat serum in PBST for 1 h at room temperature and were reacted with a mouse monoclonal anti-calbindin antibody (1:15,000; C9848; Sigma-Aldrich; RRID: AB_476894) in 5% normal goat serum-containing PBST at 4°C over two nights. After rinsing in PBST, the sections were reacted with peroxidase-labeled polymer conjugated to goat anti-mouse immunoglobulin (K4001, Dako, Carpinteria, CA, USA) for 1 h at room temperature. Calbindin immunoreactivity was visualized with a chromogenic substrate, 3,3′-diaminobenzidine (liquid DAB plus substrate chromogen system, Dako). Immunostained sections were mounted on gelatin-coated glass slides, air dried, dehydrated in ascending ethanol, cleared with xylene, and cover-slipped with a mounting medium.

#### Morphometric Analysis

The volumes of the BNSTp and the number of calbindin-immunopositive (calbindin-ir) cells in the BNSTp were measured using a light microscope equipped with a charge-coupled device camera (CX9000; MBF Bioscience, Williston, VT, USA) and a computer running Stereo Investigator software (MBF Bioscience). The outlines of the BNSTp on the left of the midline were traced to measure the volume. Calbindin-ir cells in the BNSTp were then counted using the optical fractionator method. Detailed parameters of the stereological analyses are as follows: section thickness, 30 µm; section interval, 60 µm; sampling grid size, 150 µm × 150 µm; counting frame size, 50 µm × 50 µm; dissector height, 13–16 µm; and guard zone height, 1.5 µm.

### Statistical Analysis

Two-way analysis of variance (ANOVA) was used to assess the effects of sex and age and the interaction between main factors on AVPV and BNSTp volume and the mRNA levels of target genes. The test of simple main effects was conducted when the interaction between main factors was significant in the two-way ANOVA. If there was a significant effect of age, but no significant interaction between sex and age, Bonferroni *post hoc* test was used to compare the mRNA levels of target genes between the different age groups. Differences in BNSTp volume and the number of calbindin-ir cells in the BNSTp among intact males, males subjected to prepubertal orchiectomy and cholesterol-implantation, and males subjected to prepubertal orchiectomy and testosterone implantation were analyzed with one-way ANOVA followed by Bonferroni *post hoc* test where appropriate. IBM SPSS Statistics v20.0 (IBM, Armonk, NY, USA) was used for data analyses. *P* < 0.05 was considered statistically significant. A *post hoc* power analysis was carried out after data has been collected to determine the power in this study using G*Power 3 (40), a free power analysis program.

## Results

### AVPV and BNSTp Volume on PD20–60

**Figure 1:**
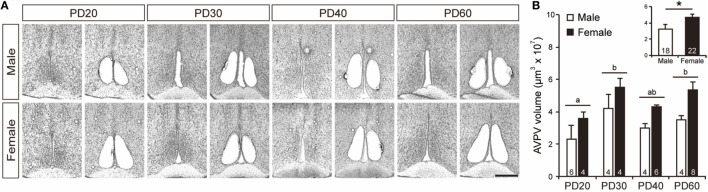
Isolation of the AVPV. **(A)** Representative photographs showing brain sections before and after the AVPV was isolated in male and female mice on PD20–60. Scale bar indicates 400 µm. **(B)** The volume of AVPV tissues isolated from the brain of male and female mice on PD20, PD30, PD40, and PD60. The inset graph in panel **(B)** indicates the average volume of the AVPV in all ages. The numbers in the columns indicate the numbers of animals. Values are the mean ± SEM. Differences in values that are significantly (*p* < 0.05) different from each other are indicated with letters. *p < 0.05.

Tissues from the AVPV were collected from cresyl fast violet-stained brain sections using the LMD system for accuracy and precision (Figure 1A). Two-way ANOVA indicated that the volume of the AVPV was significantly larger in females than in males [*F*_1, 32_ = 13.86, *p* < 0.005, power (1 − β) = 0.98; Figure 1B inset], and that it increased significantly with age from PD20 to PD60 [*F*_3, 32_ = 4.70, *p* < 0.01, power (1 − β) = 0.93; Figure 1B], although there was no significant interaction between sex and age [power (1 − β) = 0.10]. AVPV tissue volumes in PD30 mice were significantly greater (*p* < 0.05) than PD20 mice and did not differ from those in PD40 and PD60 mice.

**Figure 2:**
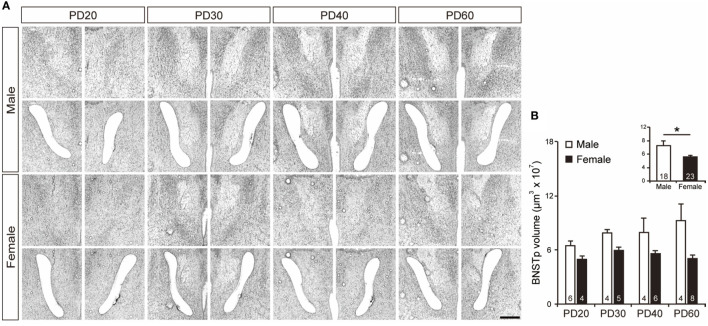
Isolation of the BNSTp. **(A)** Representative photographs showing brain sections before and after the BNSTp was isolated in male and female mice on PD20–60. Scale bar indicates 400 µm. **(B)** The volume of BNSTp tissues isolated from the brain of male and female mice on PD20, PD30, PD40, and PD60. The inset graph in panel **(B)** indicates the average volume of the BNSTp in all ages. The numbers in the columns indicate the numbers of animals. Values are the mean ± SEM. *p < 0.05.

Tissues from the BNSTp were isolated from cresyl fast violet-stained brain sections using the LMD system (Figure 2A). BNSTp volume was significantly greater in PD20–60 males than in PD20–60 females [*F*_1, 33_ = 16.33, *p* < 0.0001, power (1 − β) = 0.99; Figure 2B inset]. The main effect of age and the interaction between sex and age on BNSTp volume were not significant [power (1 − β) = 0.68 and 0.58, respectively; Figure 2B].

### Expression of *Esr1, Esr2, Cyp19a1*, and *Ar* mRNA in the AVPV on PD20–60

**Figure 3:**
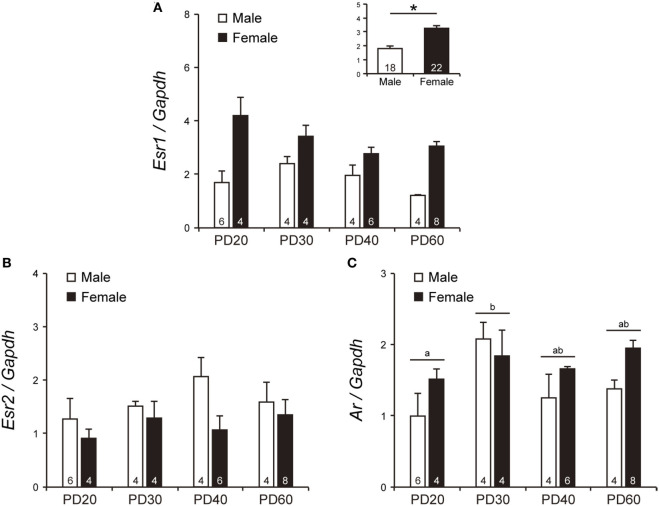
The mRNA levels of *Esr1, Esr2*, and *Ar* in the AVPV of peripubertal mice. The mRNA levels of *Esr1* **(A)**, *Esr2* **(B)**, and *Ar* **(C)** in male and female mice on PD20, PD30, PD40, and PD60. The inset graph in panel **(A)** indicates the average mRNA level of *Esr1* in all ages. The numbers in the columns indicate the numbers of animals. Values are the mean ± SEM. Differences in values that are significantly (*p* < 0.05) different from each other are indicated with letters. *p < 0.05.

Although the effect of age and interaction between sex and age on *Esr1* mRNA levels were not significant [power (1 − β) = 0.68 and 0.63, respectively], *Esr1* mRNA levels in the AVPV at PD20–60 were significantly higher in females than in males [*F*_1, 32_ = 35.75, *p* < 0.0001, power (1 − β) = 1.00; Figure 3A inset]. *Esr2* mRNA levels in the AVPV were not significantly affected by sex, age, and their interaction [power (1 − β) = 0.56, 0.25 and 0.20, respectively; Figure 3B]. *Ar* mRNA levels in the AVPV was affected significantly by age [*F*_3, 32_ = 3.03, *p* < 0.05, power (1 − β) = 0.76], but not by sex [power (1 − β) = 0.53] and the interaction between sex and age [power (1 − β) = 0.34]. *Ar* mRNA levels in the AVPV of PD30 mice were significantly higher (*p* < 0.05) than those of PD20 mice and comparable with those of PD40 and PD60 mice (Figure 3C). *Cyp19a1* mRNA in the AVPV on PD20–60 was at an undetectable level in most animals in either sex. *Cyp19a1* mRNA was detectable in one female each on PD20, PD30, and PD40, in two males at PD30, and in two males and two females at PD60, but at the limit of detection by qPCR (data not shown).

### Expression of *Esr1, Esr2, Cyp19a1*, and *Ar* mRNA in the BNSTp on PD20–60

**Figure 4:**
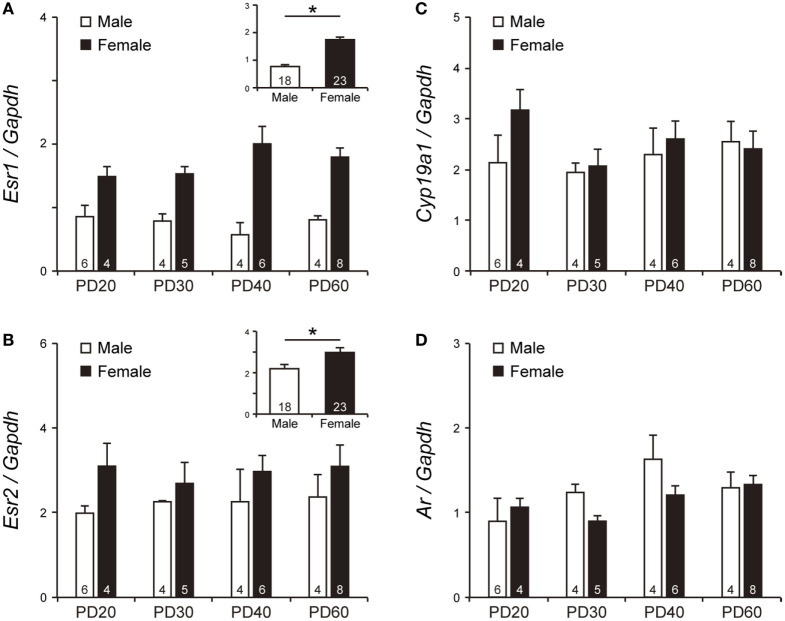
The mRNA levels of *Esr1, Esr2, Cyp19a1*, and *Ar* in the BNSTp of peripubertal mice. The mRNA levels of *Esr1* **(A)**, *Esr2* **(B)**, *Cyp19a1* **(C)**, and *Ar* **(D)** in male and female mice on PD20, PD30, PD40 and PD60. The inset graphs in panels **(A,B)** indicate the average mRNA level of *Esr1* and *Esr2* in all ages, respectively. The numbers in the columns indicate the numbers of animals. Values are the mean ± SEM. *p < 0.05.

There was a significant effect of sex [*F*_1, 33_ = 51.56, *p* < 0.0001, power (1 − β) = 1.00] on *Esr1* mRNA levels in the BNSTp during PD20–60 (Figure 4A inset). However, the *Esr1* mRNA levels were not significantly affected by age [power (1 − β) = 0.12] and by interaction between sex and age [power (1 − β) = 0.51]. *Esr2* mRNA levels in the BNSTp were significantly higher in females than in males at PD20–60 [*F*_1, 33_ = 4.35, *p* < 0.05, power (1 − β) = 0.62; Figure 4B inset], although they did not significantly change with age [power (1 − β) = 0.07] and were not affected by the interaction between sex and age [power (1 − β) = 0.08] (Figure 4B). Unlike the AVPV, the BNSTp expressed *Cyp19a1* mRNA at PD20–60 (Figure 4C). *Cyp19a1* mRNA levels in the BNSTp did not significantly change with sex and age [power (1 − β) = 0.22 and 0.24, respectively]. The interaction of sex and age was also not significant on the *Cyp19a1* mRNA levels [power (1 − β) = 0.22]. *Ar* mRNA levels in the BNSTp during PD20–60 were not significantly impacted by sex, age, and their interaction [power (1 − β) = 0.24, 0.73 and 0.40, respectively] (Figure 4D).

### Effects of Prepubertal Orchiectomy and Testosterone Replacement on the BNSTp in Male Mice

**Figure 5:**
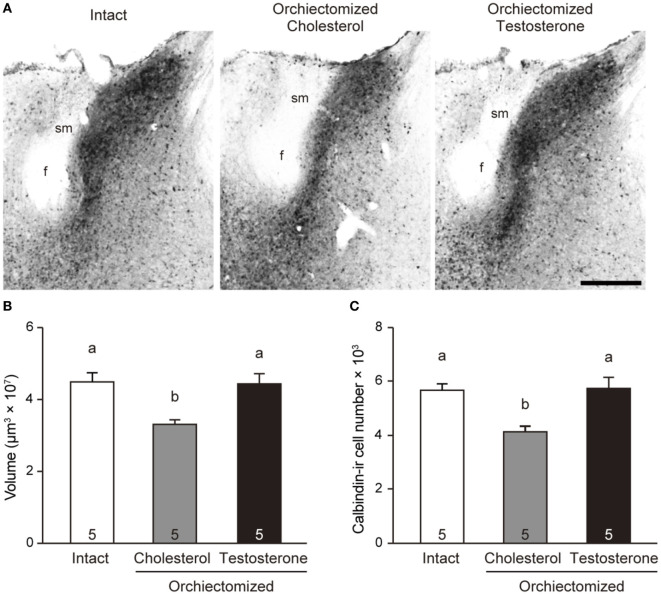
Effects of peripubertal testicular testosterone on the BNSTp in male mice. **(A)** Representative photomicrographs of brain sections from a gonadally intact adult male mouse, an adult male mouse subjected to orchiectomy and cholesterol implantation at PD20, and an adult male mouse subjected to orchiectomy and testosterone implantation at PD20. Scale bar indicates 300 µm. f, fornix; sm, stria medullaris of the thalamus. **(B)** The volume of the BNSTp in adult male mice. **(C)** The number of the calbindin-ir cells in the BNSTp of adult male mice. The numbers in the columns indicate the numbers of animals. Differences in values that are significantly (*p* < 0.05) different from each other are indicated with letters.

One-way ANOVA revealed that BNSTp volume [*F*_2, 12_ = 7.07, *p* < 0.01, power (1 − β) = 0.92] and the number of calbindin-ir cells in the BNSTp [*F*_2, 12_ = 6.98, *p* < 0.05, power (1 − β) = 0.92] of adult male mice were significantly different among the experimental groups. BNSTp volume in males that were orchiectomized and implanted with cholesterol at PD20 was significantly smaller (*p* < 0.05) than that in gonadally intact males (Figures 5A,B). However, BNSTp volume in males that were orchiectomized and implanted with testosterone at PD20 was similar to that in gonadally intact males and was significantly greater (*p* < 0.05) than that in the orchidectomzied and cholesterol-implanted males (Figures 5A,B). Likewise, the number of calbindin-ir cells in the BNSTp was significantly smaller (*p* < 0.05) in the orchiectomized and cholesterol-implanted males than in gonadally intact males (Figure 5C). The cell number in the orchiectomized and testosterone-implanted males was significantly larger (*p* < 0.05) than that in the orchiectomized and cholesterol-implanted males and was similar to that in gonadally intact males.

## Discussion

Here, we examined the mRNA levels of *Esr1, Esr2, Cyp19a1*, and *Ar* in the AVPV and BNSTp of peripubertal mice. To the best of our knowledge, this is the first study to examine these mRNA expressions to investigate the role of gonadal hormones during puberty in the formation of sexually dimorphic structures in the AVPV and BNSTp. We revealed that these genes are expressed in a sex-, age-, and region-specific manner. Especially, *Cyp19a1* mRNA was expressed in the BNSTp but not in the AVPV on PD20–60. This suggests that the AVPV and BNSTp respond differentially to testicular testosterone, which might contribute to the sexually dimorphic development of these brain structures.

### Role of Sex Steroids in the Development of the AVPV

We previously showed that the volume and neuron number in the AVPV of male mice lacking *Esr1* and *Cyp19a1* are larger than those of wild-type males and similar to those of females, although the volume and neuron number in the AVPV of male mice lacking *Esr2* are comparable to those of wild-type males (23). We further showed that the AVPV expresses *Esr1* and *Cyp19a1* in the perinatal period (23). Additionally, it has been reported that administration of testosterone to postnatal female rats reduces AVPV volume and the number of TH and kisspeptin neurons in this structure in adulthood (6, 22). In this study, we found that the AVPV expressed *Esr1* and *Esr2*, but not *Cyp19a1* during PD20–60. This finding suggests that testicular testosterone in the peripubertal period is not converted to estradiol locally in the AVPV and does not affect the AVPV of male mice *via* ESR1 and ESR2, although it is not ruled out that estradiol produced elsewhere can affect the AVPV. Taken together, it is suggested that the morphology of the male AVPV might be organized by estradiol, which is synthesized from testicular testosterone by CYP19A1 (aromatase) expressed locally in the AVPV, which signals *via* ESR1 in the perinatal, but not pubertal period. Here, we found that *Ar* mRNA was expressed in the AVPV during puberty. However, AR-mediated testosterone signaling might not be necessary for the organization of the AVPV in male mice, because the volume and neuron number in the AVPV of *Ar* KO male mice is comparable to those of wild-type male mice (23). Additionally, the AVPV in male rats appears to be not affected by prepubertal orchiectomy (33), suggesting that testicular hormones during puberty contribute minimally to the organization of the AVPV in male mice.

*Esr1* and *Esr2* were expressed in the female AVPV from PD20 to PD60. Given that ovaries begin to produce estradiol on PD7 (41), ovarian estradiol could act on the AVPV through ESR1 and ESR2 during the peripubertal period. We found here that AVPV volume increased from PD20 to PD30, and remained high on PD60, and that it was greater in female mice compared with male mice. During puberty in rats, newborn cells are incorporated into the AVPV, more so in females than in males, and this incorporation of new cells is suppressed by ovariectomy on PD20 (33). Thus, ovarian estradiol, signaling through ESR1 and/or ESR2 during puberty, might promote the generation of AVPV cells, which could contribute to the sexually dimorphic development of the AVPV.

### Role of Sex Steroids in the Development of the BNSTp

We previously reported that the masculinization of the BNSTp is disrupted in male mice lacking *Esr1, Cyp19a1*, or *Ar*, but not *Esr2* (23). We further found that the BNSTp expresses mRNAs of *Esr1* and *Cyp19a1* in the perinatal period and *Ar* mRNA in the postnatal period (23). Neonatal orchiectomy decreases the volume and number of neurons in the BNSTp of adult male rats (12), and early postnatal treatment with testosterone or estradiol increases the number of neurons in the BNSTp of adult female mice (25). These findings suggest that testosterone, acting through AR, during the postnatal period, and aromatized testosterone (estradiol), acting through ESR1, during the perinatal period help to masculinize the morphology of the BNSTp. In addition, it is thought that pubertal gonadal hormones affect the formation of the BNSTp. The BNSTp in prepubertal male mice has a greater volume and contains a larger number of calbindin neurons than the BNSTp of female mice of the same age (31, 32), and these sex differences become more pronounced over time, with a gradual increase in these parameters in males and a gradual decrease in females during puberty (31, 32). The increase in the volume and calbindin neuron number of the male BNSTp is suppressed by orchiectomy on PD20 (32). In this study, we confirmed that orchiectomy at PD20 reduced BNSTp volume and calbindin-ir cell number of the BNSTp in adult male mice. Moreover, we revealed that a compensatory treatment with testosterone could rescue the effects of orchiectomy at PD20 on the BNSTp in male mice. These findings suggest that testicular testosterone during puberty plays an important role in the formation of the male BNSTp. It was reported that the volume of the BNSTp in adult male rats is reduced by orchiectomy in adulthood (42), whereas orchiectomy in adulthood has no effect on BNSTp volume in guinea pigs (43). Therefore, the results of our current study do not exclude the notion that the formation of the male BNSTp requires testicular testosterone in the postpubertal period as well as the pubertal period. Considering that the BNSTp from PD20 to PD60 expressed the mRNAs of *Esr1, Cyp19a1*, and *Ar*, we can deduce that the masculinization of the BNSTp is affected both by testicular testosterone acting *via* AR and by estradiol, which is synthesized locally in the BNSTp by CYP19A1 from testicular testosterone and acts *via* ESR1, during the pubertal period. Thus, unlike the defeminization of the AVPV, the masuclinization of the BNSTp is likely established by testicular testosterone and estradiol not only in the perinatal period, but also in the pubertal period. In comparison, the volume and number of calbindin neurons in the BNSTp are lower in adult females than in PD20 females, and ovariectomy on PD20 does not affect the reduction in BNSTp volume or neuron number during puberty (32). Unlike the formation of the male BNSTp, the formation of the female BNSTp may be independent of ovarian hormones during puberty, despite the mRNA expression of *Esr1* and *Esr2* in this brain structure.

On PD4, the mRNA levels of *Esr1* and *Cyp19a1* in the BNSTp of *Ar* KO male mice are higher than those in wild-type male mice (23). This suggests that testosterone acting *via* AR suppresses the mRNA expression of *Esr1* and *Cyp19a1* in the male BNSTp, thereby reducing estradiol signaling in the early postnatal period. In male mice, the testosterone concentration in blood is gradually increased before puberty and it is higher levels during puberty (PD35 and PD40) (44). Therefore, if the inhibitory action of testosterone on *Esr1* and *Cyp19a1* mRNA expressions persists until puberty, the action of a large amount of testosterone *via* AR and lower ESR1 expression in the male BNSTp during puberty might stimulate male-specific development of this structure, as more testosterone would be available to bind the AR (as a lower amount would be converted to estradiol, which would also have fewer receptors to signal through). Nonetheless, it was reported that *Cyp19a1* mRNA and aromatase activity in the bed nucleus of the stria terminalis (BST) including the BNSTp of adult male rats are enhanced by testosterone actions *via* AR (45, 46). If this mechanism is functional during puberty as well as adult period, pubertal testosterone may facilitate estrogen production, followed by enhancement of estrogen signaling *via* ESR. Indeed, a marked less ESR1 in the male BNSTp than in the female BNSTp on PD20–60 may be due to a downregulation of ESR1 by a large amount of aromatized testosterone. Taken together, further experiments are required to determine the relevance between testosterone action *via* AR and estradiol action *via* ESR1 in the BNSTp during puberty in the sexually dimorphic formation of the BNSTp.

### Microscopic Isolation of Target Tissues

In this study, we isolated the AVPV and BNSTp from the brain using the LMD system. The LMD system enables precise and accurate isolation of target regions in sample tissues. This allowed us to detect sex-, age-, and region-dependent differences in the mRNA expression of genes related to sex steroid signaling. In the AVPV, we found that *Esr1* mRNA levels were greater in females at PD20–60. However, in one study, the expression of ESR1 protein in the AVPV on PD25 did not differ between the sexes (47). There might be a sex difference in the posttranscriptional stability of *Esr1* mRNA and/or the posttranslational stability of ESR1 protein. In the BNSTp, one study reported that greater *Esr1* mRNA expression in female mice is not observed at PD20, but appears by PD60 (48). Another study reported that the murine BNSTp on PD25 contains a greater number of ESR1-immunoreactive cells in females compared with males (47). In this study, although we did not find a significant difference in *Esr1* mRNA levels between sexes at each age, we observed higher mRNA levels of *Esr1* in female mice at PD20–60. Our current study showed no sex difference in *Cyp19a1* mRNA expression in the BNSTp. Another study showed that *Cyp19a1* mRNA expression in the BST is greater in male mice than in female mice (49). This discordance may be due to a difference of target area. Target area in the previous study was the BST that includes some sub-nuclei (lateral division–dorsal, intermediate, posterior and ventral parts; and medial division–anterior, ventral parts, etc.). By contrast, our current study targeted only the BNSTp (the principal nucleus of the BST posterior division).

In this study, the volume of the AVPV and BNSTp was measured. We found that volume of the AVPV was larger in female mice than that in male mice through PD20 to PD60, although the sex difference was no found at each age. It was previously reported that the sexual dimorphism in AVPV volume in rats emerges between PD30 and PD40 (2). Considering species differences, it is plausible that we correctly isolated AVPV tissues enough to detect sex- and age-related alterations. Regarding the BNSTp, we found a male-biased difference in the volume on PD20–PD60. This sex difference may be resulted from an increase in the volume of the male BNSTp during puberty as reported previously (28, 31, 32). In this study, there was a tendency that the volume of the male BNSTp increased with age. Taken together, the results that sex differences and age-related alterations in the volume of the AVPV and BNSTp could be acquired in the LMD system may guarantee the accuracy of mRNA expression analysis in the current study.

**Figure 6:**
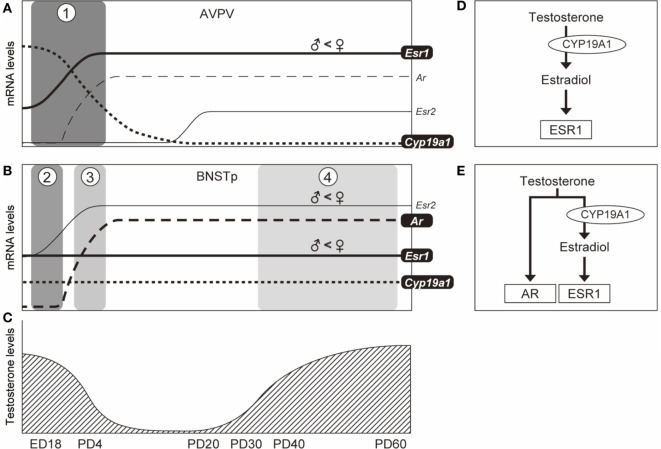
Schematic illustration of the temporal expression patterns of genes in the AVPV and BNSTp and possible testicular testosterone actions on the formation of the AVPV and BNSTp in male mice. In the AVPV [*panel* **(A)**], based upon our previous (23) and current studies, *Esr1* mRNA expression (*solid line*) increases from embryonic day 18 (ED18) to PD4 and continues in the pubertal period. *Cyp19a1* mRNA level (*dotted line*) decreases from ED18 to PD4 and is not expressed by PD20. *Esr1* and *Cyp19a1* are essential to the formation of the male AVPV, but *Esr2* and *Ar* are not necessary (23). Testicular testosterone production reaches a peak level at ED18 and decreases after birth (50), and again is increased from PD35 (44) [*panel* **(C)**]. The temporal patterns of *Esr1, Cyp19a1*, and testicular testosterone may indicate that the male AVPV is organized by estradiol (formed by the aromatization of testosterone) signaling through ESR1 during the perinatal period [*boxed phase 1 in panel* **(A)** and *panel* **(D)**]. In the BNSTp [*panel* **(B)**], based upon our previous (23) and current studies, *Esr1* mRNA (*solid line*) is expressed during the perinatal period, and is also expressed during the pubertal period. *Cyp19a1* mRNA level (*dotted line*) is stably expressed in the perinatal and pubertal periods. *Ar* mRNA (*broken line*) is not expressed in the late fetal period (ED18), but expressed in the postnatal (PD4) and pubertal periods. *Esr1, Cyp19a1*, and *AR* are requisite for the sexual differentiation of the male BNSTp (23, 26). The patterns of *Esr1, Cyp19a1, Ar*, and testicular testosterone levels may indicate that the male BNSTp is formed by the action of estradiol (formed by the aromatization of testicular testosterone) *via* ESR1 in the fetal period [*boxed phase 2 in panel* **(B)** and *panel* **(D)**], and both the action of estradiol *via* ESR1 and testicular testosterone *via* AR in the postnatal [*boxed phase 3 in panel* **(B)** and *panel* **(E)**] and pubertal [*boxed phase 4 in panel* **(B)** and *panel* **(E)**] periods.

In conclusion, pubertal testosterone is not converted to estradiol locally in the AVPV because of the absence of *Cyp19a1* mRNA. This suggests that estradiol synthesized in the AVPV itself is not able to act on the AVPV. Our previous study showed that *Esr1* and *Cyp19a1* are necessary genes for the defeminization of the AVPV, but *Esr2* and *Ar* are not, and that *Esr1* and *Cyp19a1* mRNAs are expressed in the AVPV in the perinatal period (23). Collectively, the male AVPV might be generated by estradiol (formed by the aromatization of testosterone) signaling through ESR1 during the perinatal period, but not during puberty (Figure 6). Additionally, our previous study showed that *Esr1, Cyp19a1*, and *Ar* are necessary for the masculinization of the BNSTp, but *Esr2* is not (23, 26). Furthermore, we previously reported that the BNSTp expresses *Esr1* and *Cyp19a1* mRNAs in the perinatal period and *Ar* mRNA in the postnatal period (23). In this study, we found that the BNSTp expresses *Esr1, Cyp19a1*, and *Ar* in the pubertal period. Additionally, the results of the histological analysis of the BNSTp suggest that testicular testosterone during puberty affects the formation of the BNSTp in male mice. The findings of our current study may indicate that the masculinization of the BNSTp is affected by testosterone, *via* AR, and by estradiol (formed by the aromatization of testosterone) *via* ESR1 during puberty. Thus, the sexually dimorphic development of the BNSTp in males might be accomplished by testicular testosterone in the postnatal and pubertal periods as well as by estradiol derived from testosterone in the perinatal and pubertal periods (Figure 6).

## Ethics Statement

All animal experimental procedures were approved by the Animal Care and Experimentation Committee of Saitama University and were conducted in accordance with the Guidelines for the Care and Use of Experimental Animals of Saitama University.

## Author Contributions

MK performed the experiments, analyzed the data, and prepared the manuscript. MM performed the experiments and analyzed the data. ST designed the experiments, analyzed the data, and prepared the manuscript.

## Conflict of Interest Statement

The authors declare that the research was conducted in the absence of any commercial or financial relationships that could be construed as a potential conflict of interest.
