# Correction: Temporal expression patterns of genes related to sex steroid action in sexually dimorphic nuclei during puberty

**DOI:** 10.3389/fendo.2025.1769662

**Published:** 2026-01-12

**Authors:** Moeko Kanaya, Masahiro Morishita, Shinji Tsukahara

**Affiliations:** Division of Life Science, Graduate School of Science and Engineering, Saitama University, Saitama, Japan

**Keywords:** aromatase, puberty, sex steroids, sexual differentiation, sexually dimorphic nucleus, sex steroid receptor, anteroventral periventricular nucleus, principal nucleus of the bed nucleus of the stria terminalis

In the published article, there was a mistake in the size of Silastic tube used for testosterone replacement. The size of Silastic tube was displayed as “1.02 mm inner diameter, 2.16 mm outer diameter, 10.9 mm in total length (effective length: 6.9 mm)”. The correct statement is “1.98 mm inner diameter, 3.18 mm outer diameter, 10.9 mm in total length (effective length: 6.9 mm).’’

A correction has been made to the section **Morphological Analysis of the BNSTp in Male Mice**, **Prepubertal Orchiectomy and Testosterone Replacement**, 1^st^ paragraph:

“At PD20, male mice were orchiectomized and subcutaneously implanted with a Silastic tube [1.98 mm inner diameter, 3.18 mm outer diameter, 10.9 mm in total length (effective length: 6.9 mm); Dow Corning Corporation, Midland, MI, USA] containing testosterone (Sigma-Aldrich, St. Louis, MO, USA; n = 5) or cholesterol (Wako Pure Chemical Industries, Osaka, Japan; n = 5). The testosterone implantation was designed to produce the levels of serum testosterone in young male mice (Tsukahara, unpublished data). Surgery was performed under isoflurane inhalational anesthesia (concentration, 1.5% in air; flow rate, 1 L/min). Additionally, we prepared gonadally intact males (n = 5).”

The original version of this article has been updated.

